# Early coronavirus disease 2019 (COVID-19) pandemic effects on individual-level risk for healthcare-associated infections in hospitalized patients

**DOI:** 10.1017/ice.2023.83

**Published:** 2023-12

**Authors:** Trevor S. Farthing, Ashlan Jolley, Katelin B. Nickel, Cherie Hill, Dustin Stwalley, Kimberly A. Reske, Jennie H. Kwon, Margaret A. Olsen, Jason P. Burnham, Erik R. Dubberke, Cristina Lanzas

**Affiliations:** 1 North Carolina State University, Raleigh, North Carolina; 2 Division of Infectious Diseases, Washington University, St. Louis, Missouri

## Abstract

**Objective:**

We compared the individual-level risk of hospital-onset infections with multidrug-resistant organisms (MDROs) in hospitalized patients prior to and during the coronavirus disease 2019 (COVID-19) pandemic. We also quantified the effects of COVID-19 diagnoses and intrahospital COVID-19 burden on subsequent MDRO infection risk.

**Design:**

Multicenter, retrospective, cohort study.

**Setting:**

Patient admission and clinical data were collected from 4 hospitals in the St. Louis area.

**Patients:**

Data were collected for patients admitted between January 2017 and August 2020, discharged no later than September 2020, and hospitalized ≥48 hours.

**Methods:**

Mixed-effects logistic regression models were fit to the data to estimate patients’ individual-level risk of infection with MDRO pathogens of interest during hospitalization. Adjusted odds ratios were derived from regression models to quantify the effects of the COVID-19 period, COVID-19 diagnosis, and hospital-level COVID-19 burden on individual-level hospital-onset MDRO infection probabilities.

**Results:**

We calculated adjusted odds ratios for COVID-19–era hospital-onset *Acinetobacter* spp., *P. aeruginosa* and Enterobacteriaceae spp infections. Probabilities increased 2.64 (95% confidence interval [CI], 1.22–5.73) times, 1.44 (95% CI, 1.03–2.02) times, and 1.25 (95% CI, 1.00–1.58) times relative to the prepandemic period, respectively. COVID-19 patients were 4.18 (95% CI, 1.98–8.81) times more likely to acquire hospital-onset MDRO *S. aureus* infections.

**Conclusions:**

Our results support the growing body of evidence indicating that the COVID-19 pandemic has increased hospital-onset MDRO infections.

The coronavirus disease 2019 (COVID-19) pandemic has strained healthcare organizations and disrupted delivery of care worldwide. To accommodate the surge of COVID-19 patients, healthcare facilities increased critical care capacity, reduced elective and nonemergency procedures, and modified staffing practices, and modified the use of personal protective equipment, among other strategies.^1–8^ Shortages of staff, personal protective equipment, and supplies (eg, ventilators and disinfectants) have been reported during the pandemic, along with overburdening of healthcare workers and changes in infection control guidance.^2,7,9,10^ Additional infection prevention measures, such as universal masking of patients, were also introduced to minimize severe acute respiratory coronavirus virus 2 (SARS-CoV-2) transmission within facilities.^11^ All of these changes may have influenced the effectiveness of infection prevention and control of healthcare-associated infections (HAIs).

Several studies have reported a rising incidence of various HAIs since the onset of the COVID-19 pandemic. Baker et al^1^ described the following increases, all of which correlated to surges of SARS-CoV-2 infections: 43% in catheter-associated urinary tract infections, 60% in central-line–associated bloodstream infections, 44% in methicillin-resistant *Staphylococcus aureus* (MRSA) bacteremia, and 24% in multidrug-resistant organism (MDRO) bacteremia overall.^1^ Similarly, in 2021, Weiner-Lastinger et al^8^ reported increases in the national-level standardized infection ratios for central-line–associated bloodstream infections, catheter-associated urinary tract infections, ventilator-associated events, and MRSA bacteremia in the 2020 National Healthcare Safety Network.^8^ Alternatively, some hospitals have reported reductions in HAIs, possibly due to enhanced infection control practices and heightened awareness of proper personal protective equipment use.^12,13^ Overall, previous research has focused primarily on reporting coinfections and superinfections in COVID-19 hospitalized patients.^14^ However, whether non–COVID-19 patients were also at a greater risk of HAIs during the pandemic remains unclear.

We sought to quantify whether COVID-19 pandemic changed the risk for acquiring a multidrug-resistant HAI for both COVID-19 and non–COVID-19 hospitalized patients in a multicenter retrospective cohort. We used admission and clinical data from 4 hospitals to construct mixed-effect logistic regression models and to quantify patient-level risk of acquiring an HAI associated with 1 of 8 multidrug-resistant pathogens: *Acinetobacter* spp, *Clostridioides difficile*, *Enterococcus faecium*, *Escherichia coli*, *Klebsiella pneumoniae*, *Pseudomonas aeruginosa, Staphylococcus aureus,* and general MDRO Enterobacteriaceae spp. We estimated the effect of the COVID-19 pandemic period on pathogen-specific HAI acquisition risks for hospitalized patients relative to a prepandemic period. We also quantified the effects of COVID-19 diagnoses preceding HAI onset and intrahospital COVID-19 burden during patients’ hospital stays on individual HAI risk during the pandemic period.

## Methods

### Study population

A multicenter retrospective cohort study was conducted in 4 hospitals in the St. Louis area (see Appendix 1 for detailed hospital characteristics). Admission and clinical data were collected for patients admitted between January 2017 and August 2020, discharged no later than September 2020, and hospitalized ≥48 hours. When discharged patients were readmitted to the same hospital <24 hours later, hospitalization data were concatenated into a single record. The data set consists of 260,055 unique admissions associated with 157,120 different patients. We extracted clinical and demographic data for this cohort from the BJC Medical Informatics database, including laboratory and microbiological test results, antibiotic use, and days spent in intensive care units (ICUs). Comorbidity information for this cohort was obtained using *International Classification of Disease, Tenth Revision* (ICD-10) codes with Elixhauser groupings.^15,16^ Cohort members were hospitalized for 6.83 days on average (SD, 8.30 days).

Patients with a positive culture from blood, urine, or respiratory tract specimen for any of 5 MDRO groups previously defined by the Centers for Disease Control and Prevention and described by Burnham et al^17^ were identified: MDRO Enterobacteriaceae spp, *S. aureus, P. aeruginosa, Acinetobacter* spp, and *E. faecium*. See Appendix 2 for MDRO group definitions. *C. difficile* infection was identified by positive toxin enzyme immunoassay from stool. Within the MDRO Enterobacteriaceae spp, we also separately analyzed multidrug-resistant *E. coli* and *K. pneumoniae*. We categorized infections as hospital-onset HAIs if they were first identified ≥48 hours following patient admission.^18^ COVID-19 status during admissions were based on results of SARS-CoV-2 PCR testing and/or a diagnosis code for COVID-19 (ICD-10-CM code U07.1). Medical record review was performed for admissions with a COVID-19 ICD-10-CM diagnosis code without a positive laboratory result to confirm a clinical diagnosis. To calculate the infection pressures associated with COVID-19 and HAIs, admissions for the first and last 24 days were removed. The final number of admissions used in the statistical analysis were 250,539.

### Statistical analysis

We constructed independent mixed-effects logistic regression models to estimate patients’ individual-level risk of infection with the pathogens of interest during hospitalization. Mixed-effects logistic regression models are among methods often used to characterize individual-level infection risk while considering that patients are nested within hospital.^19,20^ All analyses were done using the *lme4* R package version 1.1-27.1^21^ in R Studio version 1.4.1717 running R version 4.0.3 software. We carried out 2 sets of regression analyses, which we refer to as the “full period” and “COVID-19 period” model sets, and we describe predictor variables included in each model set in Table 1. These model sets were intended to answer 2 distinct questions. The former set identified significant (*P* ≤ .05) differences in patient-level HAI risk associated with the initial 6 months of the COVID-19 pandemic. We used “COVID-19 period” models to determine whether patients with a COVID-19 diagnosis were more or less likely to acquire these HAIs than those not diagnosed with COVID-19, and whether hospital-level COVID-19 burden modulated HAI risk for both COVID-19 and non–COVID-19 admissions. The fixed effects of interest in our models were COVID-19_period, COVID-19_diagnosis_*ix*_, and the hospital-specific COVID-19 burden during a patient stay ( 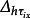 ). The following variables were also included as confounders: time spent in the hospital prior to observed infections, the relative amount of time spent on antibiotics and in intensive care units, and pathogen-specific infection pressures acting on admitted patients. See Appendix 3 (online) for additional information on our model-fitting and model selection procedures.

**Table 1.** Description of Predictor Variables Included as Fixed Effects in Mixed-Effect Logistic Regression Models

Predictor Variable	Description	Range	Associated Models	Data Type
λ 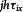	The HAI pathogen-specific colonization pressure observed in the hospital to which a patient was admitted during their LOS prior to infection diagnoses.	0–1	Full period, COVID-19 period	Proportion
Δ 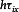	The COVID-19 burden observed in the hospital to which a patient was admitted during their LOS prior to infection diagnosis.	0–1	COVID-19 period	Proportion
COVID-19_diagnosis_j_	Binary variable describing whether patients were diagnosed with COVID-19 at admission or following admission but prior to the sample collection date for a positive sample that indicated the infection of interest. Takes the value 1 if patients met this criterion, and 0 if admissions never tested positive for SARS-COV-2 infections, never contracted the HAI of interest, or were only diagnosed with COVID-19 following the HAI diagnosis date.	0–1	COVID-19 period	Discrete
COVID-19_period	Binary variable describing whether patients were admitted on or following the date of admission for the first COVID-19 patient in the data set. Takes the value 1 if patients met this criteria, and 0 if not.	0–1	Full period	Discrete
Preceding_Antibiotic_Days_j_	For admissions with a positive bacterial culture indicating an infection with the pathogen of interest, this is the proportion of days prior to positive sample collection date during which antibiotics were administered to the patient. Defaults to 0 for admissions with no positive test during their hospital stay.	0–1	Full period, COVID-19 period	Proportion
Preceding_ICU_Days_j_	For admissions with a positive bacterial culture indicating an infection with the pathogen of interest, this is the proportion of days prior to positive sample collection date during which the patient was in an ICU. Defaults to 0 for admissions with no positive test during their hospital stay.	0–1	Full period, COVID-19 period	Proportion
Preceding_LOS_j_	Number of days individuals were hospitalized prior to sample-collection dates for positive samples indicating admissions were positive for an HAI with a pathogen of interest. For admissions that did not produce any positive sample, the value of this variable becomes the patient’s entire LOS.	2–500.125	Full period, COVID-19 period	Continuous

Note HAI, healthcare-associated infection; ICU, intensive care unit; LOS, length of stay.

This study was approved by the Washington University School of Medicine Institutional Review Board (IRB ID no. 202010057) with a waiver of informed consent.

## Results

**Figure 1.**
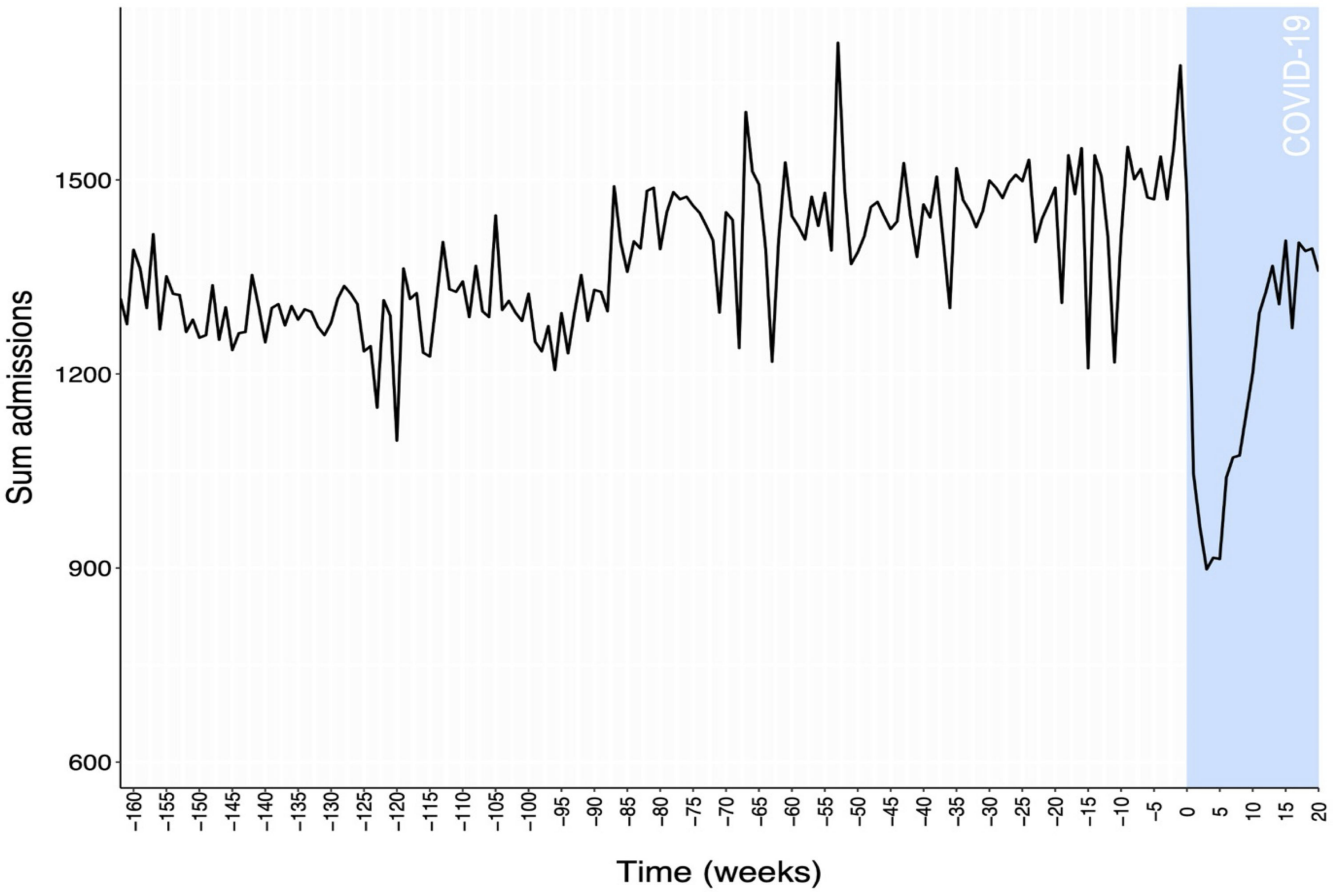
Sum admissions with lengths of stay ≥2 days admitted to study hospitals each week. Weeks containing or following the first observed COVID-19 patient are highlighted in blue and begin at week 0. Weeks preceding COVID-19 patient admissions are shown were assigned negative IDs.

**Figure 2.**
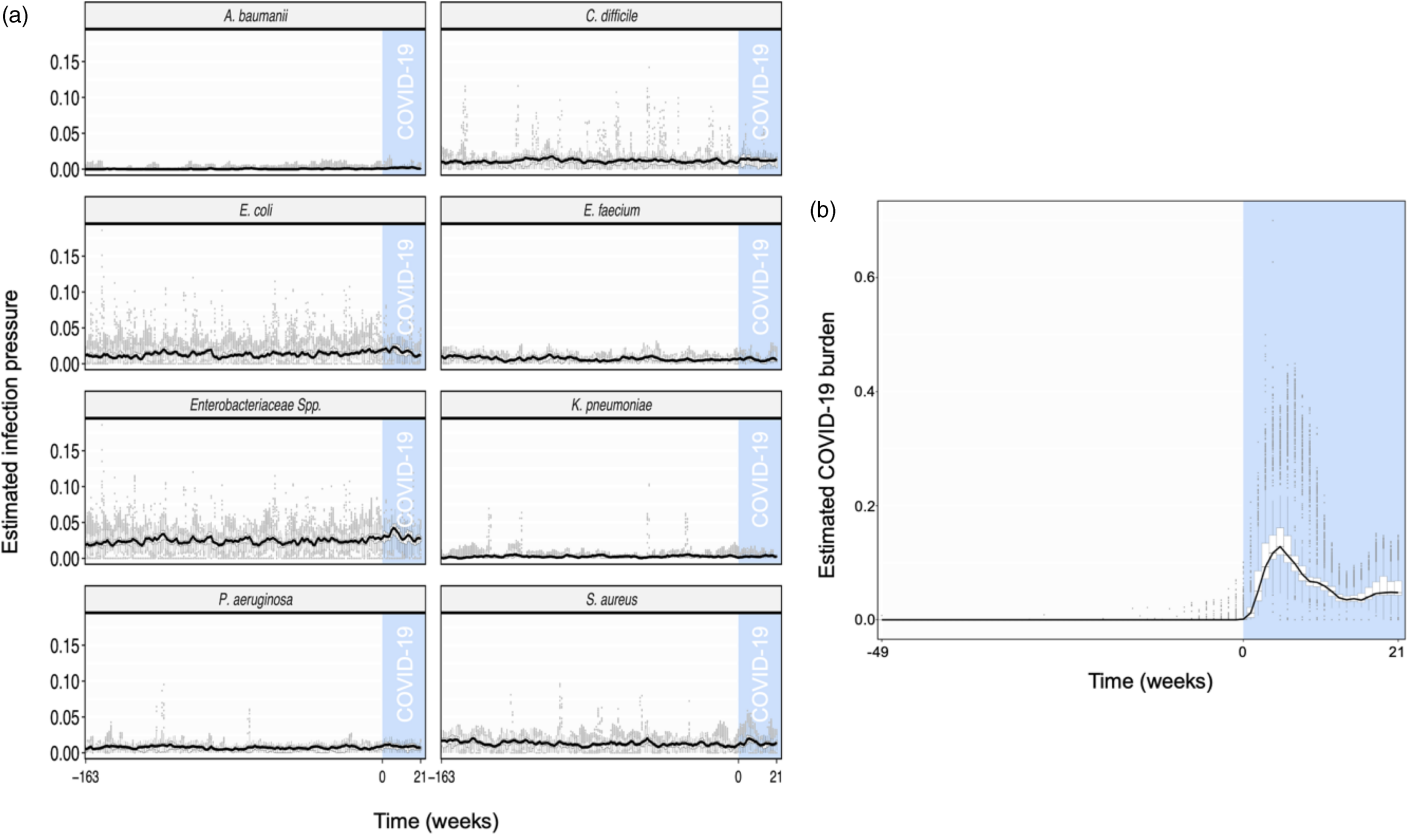
Estimated infection pressures for healthcare-associated infection (HAI) pathogens of interest (a) and COVID-19 burden (b) calculated for each individual admission record. Median estimates are shown by the black line, and weeks containing or following the first observed COVID-19 patient are highlighted in blue. All COVID-19 burden estimates for weeks preceding “Week −49” are equal to 0 and are not shown.

The numbers of admissions readily decreased during the early phase of the pandemic, followed by an increase in admissions, to numbers more similar to ranges observed during preceding years (Fig. 1). Figure 2 shows the temporal trends for the estimated infection pressures, calculated as the proportion of patient days contributed by patients with a confirm diagnosis for a given MDRO or COVID-19. There is no clear change in the trends of the MDRO infection pressures in the COVID-19 pandemic period (Fig. 2a). The pandemic period was, however, associated with a notable increase in the proportion of admitted patients presenting with SARS-CoV-2 infection during the hospital stay (Fig. 2b).

Adjusted odds ratios associated with COVID-19–period effects on HAI occurrence are given in Table 2. We observed significant COVID-19–period effects for *Acinetobacter* spp, *P. aeruginosa*, and overall Enterobacteriaceae spp HAIs, for which the odds ratios suggest hospital-onset infection probabilities increased by 2.64 (95% confidence interval [CI], 1.22–5.73) times, 1.44 (95% CI, 1.03–2.02) times, and 1.25 (95% CI, 1.00–1.58) times relative to prepandemic time points, respectively. The frequency of respiratory and urine cultures for patients remained unchanged between the prepandemic period and the pandemic period. Also, 5% of patients had a respiratory culture taken regardless of the period. Rates of urine culture were also very similar between the periods: 16% during the prepandemic period versus 17% during the pandemic period.

**Table 2.** Estimated Effects of the “COVID-19_Period” Variable on Individual-Level MDRO HAI Risk

HAI	Adjusted OR (95% CI)^a^
*Acinetobacter* spp	2.64 (1.22–5.73)
*Clostridioides difficile* ^b^	1.03 (0.82–1.29)
*Escherichia coli* ^b^	0.89 (0.61–1.25)
*Enterococcus faecium*	0.94 (0.63–1.42)
Enterobacteriaceae spp	1.25 (1.00–1.58)
*Klebsiella pneumoniae*	0.76 (0.37–1.58)
*Pseudomonas aeruginosa*	1.44 (1.03–2.02)
*Staphylococcus aureus*	1.05 (0.78–1.42)

Note. MDRO, multidrug-resistant organism; HAI, healthcare-associated infection; OR, odds ratio; CI, confidence interval.

a Adjusted ORs denoting the relative risk of pathogen-specific hospital-onset infections during COVID-19 period admissions are given. ORs for other model variables are not shown. Wald 95% CIs are listed in parentheses.

b A reduced or alternate version of the “full period” model was fit to the data.

Within the pandemic period, COVID-19 patients had both urine and respiratory cultures taken more frequently compared to non–COVID-19 patients. Also, 16% of COVID-19 patients had respiratory cultures versus 4% of non–COVID-19 patients during the pandemic period. Furthermore, 27% of COVID-19 patients had urine cultures versus 16% of non–COVID-19 patients during the pandemic period. Table 3 lists odds ratios associated with effects of COVID-19 diagnoses immediately preceding HAIs and hospital COVID-19 burden ( 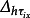 ) during patient stays. Overall, a prior COVID-19 diagnosis and the hospital COVID-19 burden were not associated with an increase in patient-level risk for MDRO infection. The only exception was that COVID-19 patients were 4.18 (95% CI, 1.98–8.81) times more likely to acquire a hospital-onset MDRO *S. aureus* infection. Interestingly, half of our models estimated that COVID-19 burden had protective effects on infection probabilities (ie, odds ratio < 1); however, these effects were not significant at an α level of 0.05. Estimates for all predictors included in all the models are reported in Appendix 3 (online). Overall, number of prior days in the ICU was a significant variable for all HAI risk models, and prior days of antibiotics was also significant for most models.

**Table 3.** Estimated Effects of COVID-19 Diagnoses and COVID-19 Burden (Δ 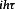 ) on Individual-Level MDRO HAI Risk

HAI	Variable of Interest	Adjusted OR (95% CI)^a^
*Acinetobacter* spp^a^	Δ 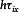	1.12 (0.71–1.72)
	COVID-19_diagnosis	1.29 (0.06–8.97)
*Clostridioides difficile*	Δ 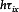	0.88 (0.73–1.07)
	COVID-19_diagnosis	1.07 (0.40–2.83)
*Escherichia coli* ^b^	Δ 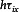	1.08 (0.85–1.37)
	COVID-19_diagnosis	1.40 (0.41–3.95)
*Enterococcus faecium* ^b^	Δ 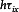	1.00 (0.75–1.29)
	COVID-19_diagnosis	1.33 (0.19–5.15)
Enterobacteriaceae spp^b^	Δ 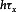	0.97 (0.86–01.08)
	COVID-19_diagnosis	1.74 (0.97–2.98)
*Klebsiella pneumoniae* ^b^	Δ 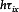	0.89 (0.52–1.33)
	COVID-19_diagnosis	1.26 (0.06–9.05)
*Pseudomonas aeruginosa* ^b^	Δ 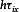	0.86 (0.66–1.05)
	COVID-19_diagnosis	2.22 (0.86–5.14)
*Staphylococcus aureus*	Δ 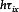	1.03 (0.87–1.21)
	COVID-19_diagnosis	4.18 (1.98–8.81)

Note. MDRO, multidrug-resistant organism; HAI, healthcare-associated infection; OR, odds ratio; CI, confidence interval.

a Adjusted ORs denoting the relative risk attributable to 1-unit increases of these variables on pathogen-specific hospital-onset infection probability are given in the Table. ORs for other model variables are not shown. Wald 95% CIs are given in parentheses.

b A reduced or alternate version of the “COVID-19 period” model was fit to the data.

## Discussion

We constructed mixed-effect logistic regression models to estimate COVID-19 effects on the patient-level risk for acquiring 8 MDRO HAIs. Patients admitted to hospitals between March and August 2020 (ie, during the COVID-19 pandemic period) were more likely to become infected with MDRO *P. aeruginosa* and Enterobacteriaceae spp and *Acinetobacter* spp than their prepandemic counterparts, but we did not observe significant period effects for other MDRO HAIs. The increase on infections with these pathogens was not linked to an increase in the frequency of culture during the pandemic period or COVID-19 diagnosis. However, MDRO *P. aeruginosa* and Enterobacteriaceae spp and *Acinetobacter* spp infections were associated with the preceding duration of stay in the ICU, suggesting that increased risk to infection may reflect a sicker population during the COVID-19 pandemic period. Prior studies have associated the increased HAI risks with a sicker patient population requiring ICU care, more frequent use of invasive devices, longer courses of antibiotics, and longer stays.^22^ In our study, patients during the prepandemic period and non–COVID-19 patients in the pandemic period were similar, with comparable lengths of stays, age, comorbidities, and hospital mortality rates. However, there was a slightly increase in the percentage of ICU admissions for non–COVID-19 patients in some hospitals during the pandemic period (Table A1-2). Overall, COVID-19 patients were more severely ill, with longer lengths of stay, a higher ICU admission rate, and a higher hospital mortality rate (Table A1-2). In our COVID-19–period models, we accounted for the effects of patient susceptibility by including COVID-19 diagnosis and preceding days on antibiotics and in the ICU (Table 1). Additionally, we included intrahospital COVID-19 burden during the patient’s hospital stay as a variable to account for the potential indirect effects related to the burden of COVID-19 patients in the facilities, such as staff shortage and burnout or infection control changes (Table 1). Proportion of prior days in the ICU was a significant variable for all the HAI risk models, and proportion of prior days on antibiotics was also significant for most models (Appendices 2 and 3). However, COVID-19 burden was not significant in any of the HAI models (Table 3). Statistical models by Baker et al^1^ and standardized infection ratios by Weiner-Lastinger et al^8^ suggest that local COVID-19 burden was positively correlated with hospital-onset infections at the hospital level. These studies did not quantify the effects of COVID-19 burden at the individual level of risk, however. Our findings suggest that the signal observed in previous studies may capture effects of COVID-19–mediated changes in admitted patients’ hospital stay characteristics (eg, length of stay in ICUs) rather than facility-mediated COVID-19 infection effects, such as staff or infection control changes.

Our findings also indicated that having a positive COVID-19 diagnosis increased the probability of subsequently developing a hospital-onset MDRO *S. aureus* infection, but we could not conclude the same for other HAIs. In general, previous studies have indicated that COVID-19 patients are at increased risk, relative to non–COVID-19 patients, for hospital-onset MDRO HAIs.^22–28^ Superinfection with MDRO *S. aureus* has been described in previous reports describing HAI incidence in COVID-19 patients.^8,14^ Superinfection with MDRO *S. aureus* have been directly attributed to the management of COVID-19 patients, which often included used of catheters, intubation, and corticosteroid use.

Although we did not report an increased risk on other HAIs with COVID-19 diagnosis, others have reported increases in other HAIs. Garcia-Vidal et al^26^ noted that *E. coli* and *P. aeruginosa* infections were common in COVID-19 patients. Baskaran et al^24^ identified *E. coli* and *K. pneumoniae* as representing the majority of COVID-19–related HAIs. Study populations in these previous studies were comprised solely of critically ill COVID-19 patients or COVID-19 patients admitted to ICUs.^24,25,27^ Thus, the distribution of HAIs affecting these populations may differ from ours. Our data set contained all ≥48-hour hospital admissions, and we did not limit our analyses to ICU admissions.

The main strengths of our study are the multisite nature of the study and that our cohort represented all hospitalized patients, including non–COVID-19 and non-ICU patients. The primary limitation of this study is that the scope of our data were restricted to patient-level post–hospital-admission attributes. Because we were unable to characterize hospital staffing and infection control policy changes, we ultimately lacked the ability to conclusively identify specific causes of COVID-19–related HAI risk modifications. Additionally, we defined the beginning of the COVID-19 period as the time point when the first recorded COVID-19 patient was admitted to one of the hospitals in this study. It is possible that patients infected with SARS-CoV-2 were admitted prior to this date, but their condition went undiscovered due to lack of testing and relative obscurity of the disease.

Our findings suggest that the COVID-19 pandemic has indirectly affected MDRO pathogen transmission in hospitals and that COVID-19 diagnosis may be related to MDRO *S. aureus* infection risk. These findings can be used to calibrate mechanistic transmission models for simulating HAI incidence, like that presented by Stephenson et al,^29^ and can help to illuminate causative HAI transmission mechanisms in this system. Our work will contribute to the development of effective interventions for preventing HAIs and MDRO transmission in hospitals.

## Supplementary material

For supplementary material accompanying this paper visit https://doi.org/10.1017/ice.2023.83. click here to view supplementary material

## References

[1] Baker MA , Sands KE , Huang SS , et al. The impact of coronavirus disease 2019 (COVID-19) on healthcare-associated infections. Clin Infect Dis 2021:ciab688. 10.1093/cid/ciab688 PMC8385925 34370014

[2] Cheeyandira A. The effects of COVID-19 pandemic on the provision of urgent surgery: a perspective from the USA. J Surg Case Rep 2020;2020:rjaa109. 32346470 10.1093/jscr/rjaa109 PMC7176477

[3] Fakih MG , Bufalino A , Sturm L , et al. Coronavirus disease 2019 (COVID-19) pandemic, central-line–associated bloodstream infection (CLABSI), and catheter-associated urinary tract infection (CAUTI): the urgent need to refocus on hardwiring prevention efforts. Infect Control Hosp Epidemiol 2022;43:26–31. 33602361 10.1017/ice.2021.70 PMC8007950

[4] McMullen KM , Smith BA , Rebmann T. Impact of SARS-CoV-2 on hospital-acquired infection rates in the United States: predictions and early results. Am J Infect Control 2009;37:A14. 10.1016/j.ajic.2020.06.209 PMC7329659 32621857

[5] Pascale R , Bussini L , Gaibani P , et al. Carbapenem-resistant bacteria in an intensive care unit during the coronavirus disease 2019 (COVID-19) pandemic: a multicenter before-and-after cross-sectional study. Infect Control Hosp Epidemiol 2022;43:461–466. 33858547 10.1017/ice.2021.144 PMC8365044

[6] Rawson TM , Moore LSP , Castro-Sanchez E , et al. COVID-19 and the potential long-term impact on antimicrobial resistance. J Antimicrob Chemother 2020;75:1681–1684. 32433765 10.1093/jac/dkaa194 PMC7314000

[7] Rebmann T , Alvino RT , Holdsworth JE. Availability and crisis standards of care for personal protective equipment during fall 2020 of the COVID-19 pandemic: a national study by the APIC COVID-19 task force. Am J Infect Control 2009;37:A14. 10.1016/j.ajic.2021.03.015 PMC7997145 33775741

[8] Weiner-Lastinger L , Pattabiraman V , Konnor RY , et al. The impact of coronavirus disease 2019 (COVID-19) on healthcare-associated infections in 2020: a summary of data reported to the national healthcare safety network. Infect Control Hosp Epidemiol 2022;43:12–25. 34473013 10.1017/ice.2021.362

[9] Gohar B , Larivière M , Nowrouzi-Kia B. Sickness absence in healthcare workers during the COVID-19 pandemic. Occup Med (Lond) 2020;70:338–342. 32449751 10.1093/occmed/kqaa093 PMC7313824

[10] Rebmann T , Alvino RT , Mazzara RL , Sandcork J. Infection preventionists’ experiences during the first nine months of the COVID-19 pandemic: findings from focus groups conducted with Association of Professionals in Infection Control & Epidemiology (APIC) members. Am J Infect Control 2021;49:1093–1098. 34454681 10.1016/j.ajic.2021.07.003 PMC8387098

[11] Wang X , Ferro EG , Zhou G , Hashimoto D , Bhatt DL. Association between universal masking in a health care system and SARS-CoV-2 positivity among healthcare workers. JAMA 2020;324:703–704. 32663246 10.1001/jama.2020.12897 PMC7362190

[12] Bentivegna E , Luciani M , Arcari L , Santino I , Simmaco M , Martelletti P. Reduction of multidrug-resistant (MDR) bacterial infections during the COVID-19 pandemic: a retrospective study. Int J Envir Res Public Health 2021;18:1003. 10.3390/ijerph18031003 PMC7908142 33498701

[13] Cole J , Barnard E. The impact of COVID-19 pandemic on healthcare-acquired infections with multidrug-resistant organisms. Am J Infect Control 2009;37:A14. 10.1016/j.ajic.2020.09.013 PMC7529600 33011335

[14] Kubin CJ , McConville TH , Dietz D , et al. Characterization of bacterial and fungal infections in hospitalized patients with coronavirus disease 2019 and factors associated with health care-associated infections. Open Forum Infect Dis 2021;8:ofab201. 34099978 10.1093/ofid/ofab201 PMC8135866

[15] HCUP Elixhauser comorbidity software - Healthcare cost and utilization project (HCUP). Agency for Healthcare Research and Quality website. https://hcup-us.ahrq.gov/toolssoftware/comorbidity/comorbidity.jsp. Accessed June 2023.

[16] Elixhauser A , Steiner C , Harris DR , Coffey RM. Comorbidity measures for use with administrative data. Med Care 1998;36:8–27. 9431328 10.1097/00005650-199801000-00004

[17] Burnham JP , Olsen MA , Stwalley D , Kwon JH , Babcock HM , Kollef MH. Infectious diseases consultation reduces 30-day and 1-year all-cause mortality for multidrug-resistant organism infections. Open Forum Infect Dis 2018;5:ofy026. 29577058 10.1093/ofid/ofy026 PMC5852998

[18] National Healthcare Safety Network. Patient safety component manual. US Centers for Disease Control and Prevention website. https://www.cdc.gov/nhsn/pdfs/pscmanual/pcsmanual_current.pdf. Published 2022. Accessed June 2023.

[19] Gomila A , Carratalà J , Eliakim-Raz N , et al. Risk factors and prognosis of complicated urinary tract infections caused by *Pseudomonas aeruginosa* in hospitalized patients: a retrospective multicenter cohort study. Infect Drug Resist 2018;2018:2571–2581. 10.2147/IDR.S185753 PMC6302800 30588040

[20] Clements ACA , Tong ENC , Morton AP , Whitby M. Risk stratification for surgical site infections in Australia: evaluation of the US National Nosocomial Infection Surveillance risk index. J Hosp Infect 2007;66:148–155. 17493705 10.1016/j.jhin.2007.02.019

[21] Bates D , Mächler M , Bolker B , Walker S. Fitting linear mixed-effects models using lme4. J Stat Soft 2015;67:1.

[22] COVID-19: US impact on antimicrobial resistance, special report 2022. US Centers for Disease Control and Prevention website. https://www.cdc.gov/drugresistance/pdf/covid19-impact-report-508.pdf. Published 2022. Accessed June 2023.

[23] Isonne C , Baccolini V , Migliara G , et al. Comparing the occurrence of healthcare-associated infections in patients with and without COVID-19 hospitalized during the pandemic: a 16-month retrospective cohort study in a hospital intensive care unit. J Clin Med 2022;11:1446. 35268538 10.3390/jcm11051446 PMC8910983

[24] Baskaran V , Lawrence H , Lansbury LE , et al. Coinfection in critically ill patients with COVID-19: an observational cohort study from England. J Med Microbiol 2021;70:001350. 33861190 10.1099/jmm.0.001350 PMC8289210

[25] Blonz G , Kouatchet A , Chudeau N , et al. Epidemiology and microbiology of ventilator-associated pneumonia in COVID-19 patients: a multicenter retrospective study in 188 patients in an un-inundated French region. Crit Care 2021;25:72. 33602296 10.1186/s13054-021-03493-w PMC7891465

[26] Garcia-Vidal C , Sanjuan G , Moreno-García E , et al. Incidence of coinfections and superinfections in hospitalized patients with COVID-19: a retrospective cohort study. Clin Microbiol Infect 2021;17:83–88. 10.1016/j.cmi.2020.07.041 PMC7836762 32745596

[27] Grasselli G , Scaravilli V , Mangioni D , et al. Hospital-acquired infections in critically ill patients with COVID-19. Chest 2021;160:454–465. 33857475 10.1016/j.chest.2021.04.002 PMC8056844

[28] Martinez-Guerra B , Gonzalez-Lara M , de-Leon-Cividanes NA , et al. Antimicrobial resistance patterns and antibiotic use during hospital conversion in the COVID-19 pandemic. Antibiotics 2021;10:182. 33670316 10.3390/antibiotics10020182 PMC7917840

[29] Stephenson B , Lanzas C , Lenhart S , et al. Comparing intervention strategies for reducing clostridioides difficile transmission in acute healthcare settings: an agent-based modeling study. BMC Infect Dis 2020;20:799. 33115427 10.1186/s12879-020-05501-w PMC7594474

