# Supplementary material for: Early coronavirus disease 2019 (COVID-19) pandemic effects on individual-level risk for healthcare-associated infections in hospitalized patients
Source: Infect Control Hosp Epidemiol. 2023 Jun 29;44(12):1966–71. doi: 10.1017/ice.2023.83 (PMC10755158; doi:10.1017/ice.2023.83)
Supplement: Supplementary file 1 [file S0899823X23000831sup001.docx]

**APPENDIX 1 – Hospital characteristics**

Table A1-1: Populations served and demographics of COVID-19 cases for service areas of 4 St. Louis, MO hospitals from which data were collected (A – D).

|  | **A** | **B** | **C** | **D** |
| --- | --- | --- | --- | --- |
| Beds | 1260 | 456 | 482 | 68 |
| Population served |  |  |  |  |
| Median household income | $28,478 | $22,604 | $92,664 | $99,100 |
| Population density per square mile | 5,157 | 2,512 | 926 | 1,736 |
| White | 46.2% | 22.5% | 84.7% | 79.9% |
| Black | 46.9% | 75.5% | 1.7% | 7.2% |
| Age >65 | 12.6% | 16.8% | 29.3% | 20.7% |
| Bachelor’s degree or higher | 35.0% | 14.6% | 74.4% | 70.0% |

Table A1-2. Hospital admission summary statistics. Reported comorbidity statistics are derived from Elixhauser category values for the population.^15,16^ *The COVID-19 pandemic time period is defined here as “on or following the first day a SARS-CoV-2 positive patient was admitted,” and we report statistic values for admissions associated with positive SARS-CoV-2 diagnoses separately from others. ^†^Standard deviations are given in parentheses. ^§^Time-period specific percentages of total or sub-populations are given in parentheses.

|  |  | Pre-COVID-19 pandemic | COVID-19 pandemic time period*  (no SARS-CoV-2 positive diagnosis) | COVID-19 pandemic time period*  (SARS-CoV-2 positive diagnosis) | Total |
| --- | --- | --- | --- | --- | --- |
| **Number of days** |  | 1,141 | 149 | 149 | 1,290 |
| **Number of admissions** | *Hospital A* | 133,450 | 15,061 | 674 | 149,185 |
|  | *Hospital B* | 33,820 | 3,395 | 430 | 37,645 |
|  | *Hospital C* | 53,206 | 5,230 | 236 | 58,672 |
|  | *Hospital D* | 4,516 | 498 | 23 | 5037 |
|  | *All hospitals* | 224,992 | 24,184 | 1,363 | 250,539 |
| **Number of patients** | *Hospital A* | 85,018 | 12,246 | 626 | 93,551 |
|  | *Hospital B* | 19,955 | 2,810 | 416 | 21,866 |
|  | *Hospital C* | 37,628 | 4,631 | 230 | 41,057 |
|  | *Hospital D* | 3,806 | 461 | 23 | 4,207 |
|  | *All hospitals* | 139,128 | 19,703 | 1,272 | 152,200 |
| **Mean length of stay – days, (SD)** | *Hospital A* | 7.61 (9.74)^†^ | 7.72 (8.97)^†^ | 12.36 (13.34)^†^ | 7.64 (9.69)^†^ |
|  | *Hospital B* | 6.29 (6.61)^†^ | 6.11 (5.40)^†^ | 11.71 (10.87)^†^ | 6.34 (6.60)^†^ |
|  | *Hospital C* | 5.37 (4.95)^†^ | 5.26 (4.59)^†^ | 12.45 (11.35)^†^ | 5.39 (4.98)^†^ |
|  | *Hospital D* | 3.65 (2.37)^†^ | 4.09 (2.61)^†^ | 6.69 (3.71)^†^ | 3.70 (2.42)^†^ |
|  | *All hospitals* | 6.80 (8.36)^†^ | 6.89 (7.75)^†^ | 12.07 (12.17)^†^ | 6.84 (8.34)^†^ |
| ***Sex*** |  |  |  |  |  |
| **Female** | *Hospital A* | 69,777 (52%)^§^ | 7,622 (51%)^§^ | 300 (45%)^§^ | 77,699 (52%)^§^ |
|  | *Hospital B* | 18,334 (54%)^§^ | 1,803 (53%)^§^ | 235 (55%)^§^ | 20,372 (54%)^§^ |
|  | *Hospital C* | 34,434 (65%)^§^ | 3,321 (63%)^§^ | 124 (53%)^§^ | 378,79 (65%)^§^ |
|  | *Hospital D* | 2,795 (62%)^§^ | 302 (61%)^§^ | 11 (48%)^§^ | 3,108 (62%)^§^ |
|  | *All hospitals* | 125,340 (56%)^§^ | 13,048 (54%)^§^ | 670 (49%)^§^ | 139,058 (56%)^§^ |
| **Male** | *Hospital A* | 63,673 (48%)^§^ | 7,439 (49%)^§^ | 374 (55%)^§^ | 71,486 (48%)^§^ |
|  | *Hospital B* | 15,486 (46%)^§^ | 1,592 (47%)^§^ | 195 (45%)^§^ | 17,273 (46%)^§^ |
|  | *Hospital C* | 18,772 (35%)^§^ | 1,909 (37%)^§^ | 112 (47%)^§^ | 20,793 (35%)^§^ |
|  | *Hospital D* | 1,721 (38%)^§^ | 196 (39%)^§^ | 12 (52%)^§^ | 1,929 (38%)^§^ |
|  | *All hospitals* | 99,652 (44%)^§^ | 11,136 (46%)^§^ | 693 (51%)^§^ | 111,481 (44%)^§^ |
| ***Age*** |  |  |  |  |  |
| **Mean age (SD)** | *Hospital A* | 55.40 (18.31)^†^ | 54.80 (18.32)^†^ | 59.96 (17.34)^†^ | 55.36 (18.31)^†^ |
|  | *Hospital B* | 61.59 (17.07)^†^ | 61.37 (17.15)^†^ | 64.11 (17.68)^†^ | 61.60 (17.09)^†^ |
|  | *Hospital C* | 60.65 (20.89)^†^ | 59.90 (20.76)^†^ | 64.34 (17.94)^†^ | 60.60 (20.87)^†^ |
|  | *Hospital D* | 58.69 (17.25)^†^ | 56.60 (19.00)^†^ | 66.43 (16.42)^†^ | 58.51 (17.44)^†^ |
|  | *All hospitals* | 57.64 (18.96)^†^ | 56.86 (18.93)^†^ | 62.14 (17.65)^†^ | 57.59 (18.95)^†^ |
| **Adult (18-64 years)** | *Hospital A* | 86,319 (65%)^§^ | 9,876 (66%)^§^ | 385 (57%)^§^ | 96,571 (65%)^§^ |
|  | *Hospital B* | 18,326 (54%)^§^ | 1,819 (54%)^§^ | 210 (49%)^§^ | 20,355 (54%)^§^ |
|  | *Hospital C* | 26,545 (50%)^§^ | 2,625 (50%)^§^ | 119 (50%)^§^ | 29,289 (50%)^§^ |
|  | *Hospital D* | 2,674 (59%)^§^ | 305 (61%)^§^ | 10 (43%)^§^ | 2,989 (59%)^§^ |
|  | *All hospitals* | 133,864 (59%)^§^ | 14,616 (60%)^§^ | 724 (53%)^§^ | 149,204 (60%)^§^ |
| **Elderly (≥65 years)** | *Hospital A* | 47,131 (35%)^§^ | 5,194 (34%)^§^ | 289 (43%)^§^ | 52,614 (35%)^§^ |
|  | *Hospital B* | 15,494 (46%)^§^ | 1,576 (46%)^§^ | 220 (51%)^§^ | 17,290 (46%)^§^ |
|  | *Hospital C* | 26,661 (50%)^§^ | 2,605 (50%)^§^ | 117 (50%)^§^ | 29,383 (50%)^§^ |
|  | *Hospital D* | 1,842 (41%)^§^ | 193 (39%)^§^ | 13 (57%)^§^ | 2,048 (41%)^§^ |
|  | *All hospitals* | 91,128 (41%)^§^ | 9,568 (40%)^§^ | 639 (47%)^§^ | 101,335 (40%)^§^ |
| ***Comorbidities*** |  |  |  |  |  |
| **Chronic kidney disease** | *Hospital A* | 26,942 (20%)^§^ | 3,237 (21%)^§^ | 224 (33%)^§^ | 30,403 (20%)^§^ |
|  | *Hospital B* | 10,755 (32%)^§^ | 1,193 (35%)^§^ | 172 (40%)^§^ | 12,120 (32%)^§^ |
|  | *Hospital C* | 11,684 (22%)^§^ | 1,160 (22%)^§^ | 71 (30%)^§^ | 12,915 (22%)^§^ |
|  | *Hospital D* | 429 (9%)^§^ | 59 (12%)^§^ | 8 (35%)^§^ | 496 (10%)^§^ |
|  | *All hospitals* | 49,810 (22%)^§^ | 5,649 (23%)^§^ | 475 (35%)^§^ | 55,934 (22%)^§^ |
| **Chronic pulmonary disease** | *Hospital A* | 31,888 (24%)^§^ | 3,764 (25%)^§^ | 185 (27%)^§^ | 35,837 (24%)^§^ |
|  | *Hospital B* | 12,772 (38%)^§^ | 1,226 (36%)^§^ | 134 (31%)^§^ | 14,132 (38%)^§^ |
|  | *Hospital C* | 12,371 (23%)^§^ | 1,219 (23%)^§^ | 55 (23%)^§^ | 13,645 (23%)^§^ |
|  | *Hospital D* | 666 (15%)^§^ | 92 (18%)^§^ | 2 (9%)^§^ | 760 (15%)^§^ |
|  | *All hospitals* | 57,697 (26%)^§^ | 6,301 (26%)^§^ | 376 (28%)^§^ | 64,374 (26%)^§^ |
| **Diabetes – complicated or uncomplicated** | *Hospital A* | 36,728 (28%)^§^ | 4,149 (28%)^§^ | 298 (44%)^§^ | 41,175 (28%)^§^ |
|  | *Hospital B* | 14,783 (44%)^§^ | 1,548 (46%)^§^ | 207 (48%)^§^ | 16,538 (44%)^§^ |
|  | *Hospital C* | 14,579 (27%)^§^ | 1,451 (28%)^§^ | 101 (43%)^§^ | 16,131 (28%)^§^ |
|  | *Hospital D* | 818 (18%)^§^ | 120 (24%)^§^ | 7 (30%)^§^ | 945 (19%)^§^ |
|  | *All hospitals* | 66,908 (30%)^§^ | 7,268 (30%)^§^ | 613 (45%)^§^ | 74,789 (30%)^§^ |
| **Hypertension – complicated or uncomplicated** | *Hospital A* | 78,363 (59%)^§^ | 9,169 (61%)^§^ | 505 (75%)^§^ | 88,037 (59%)^§^ |
|  | *Hospital B* | 27,218 (80%)^§^ | 2,826 (83%)^§^ | 364 (85%)^§^ | 30,408 (81%)^§^ |
|  | *Hospital C* | 32,128 (60%)^§^ | 3,221 (62%)^§^ | 168 (71%)^§^ | 35,517 (61%)^§^ |
|  | *Hospital D* | 1,883 (42%)^§^ | 285 (57%)^§^ | 15 (65%)^§^ | 2,183 (43%)^§^ |
|  | *All hospitals* | 139,592 (62%)^§^ | 15,501 (64%)^§^ | 1,052 (77%)^§^ | 156,145 (62%)^§^ |
| **Liver disease** | *Hospital A* | 10,810 (8%)^§^ | 1,387 (9%)^§^ | 46 (7%)^§^ | 12,243 (8%)^§^ |
|  | *Hospital B* | 2,271 (7%)^§^ | 276 (8%)^§^ | 27 (6%)^§^ | 2,574 (7%)^§^ |
|  | *Hospital C* | 2,923 (5%)^§^ | 308 (6%)^§^ | 10 (4%)^§^ | 3,241 (6%)^§^ |
|  | *Hospital D* | 352 (8%)^§^ | 30 (6%)^§^ | 1 (4%)^§^ | 383 (8%)^§^ |
|  | *All hospitals* | 16,356 (7%)^§^ | 2,001 (8%)^§^ | 84 (6%)^§^ | 18,441 (7%)^§^ |
| **Metastatic cancer** | *Hospital A* | 12,117 (9%)^§^ | 1,472 (10%)^§^ | 24 (4%)^§^ | 13,613 (9%)^§^ |
|  | *Hospital B* | 1,213 (4%)^§^ | 126 (4%)^§^ | 6 (1%)^§^ | 1,345 (4%)^§^ |
|  | *Hospital C* | 2,257 (4%)^§^ | 225 (4%)^§^ | 6 (3%)^§^ | 2,488 (4%)^§^ |
|  | *Hospital D* | 357 (8%)^§^ | 45 (9%)^§^ | 2 (9%)^§^ | 404 (8%)^§^ |
|  | *All hospitals* | 15,944 (7%)^§^ | 1,868 (8%)^§^ | 38 (3%)^§^ | 17,850 (7%)^§^ |
| ***In-hospital activity*** |  |  |  |  |  |
| **Total admissions to ICU** | *Hospital A* | 30,129 (23%)^§^ | 3,433 (23%)^§^ | 317 (47%)^§^ | 33,879 (23%)^§^ |
|  | *Hospital B* | 4,285 (13%)^§^ | 605 (18%)^§^ | 190 (44%)^§^ | 5,080 (13%)^§^ |
|  | *Hospital C* | 7,876 (15%)^§^ | 817 (16%)^§^ | 81 (34%)^§^ | 8,774 (15%)^§^ |
|  | *Hospital D* | 497 (11%)^§^ | 0 (0%)^§^ | 0 (0%)^§^ | 497 (10%)^§^ |
|  | *All hospitals* | 42,787 (19%)^§^ | 4,855 (20%)^§^ | 588 (43%)^§^ | 48,230 (19%)^§^ |
| **Died in hospital** | *Hospital A* | 23,020 (17%)^§^ | 1,571 (10%)^§^ | 148 (22%)^§^ | 24,739 (17%)^§^ |
|  | *Hospital B* | 5,831 (17%)^§^ | 410 (12%)^§^ | 97 (23%)^§^ | 6,338 (17%)^§^ |
|  | *Hospital C* | 7,538 (14%)^§^ | 439 (8%)^§^ | 50 (21%)^§^ | 8,027 (14%)^§^ |
|  | *Hospital D* | 674 (15%)^§^ | 32 (6%)^§^ | 4 (17%)^§^ | 710 (14%)^§^ |
|  | *All hospitals* | 37,063 (16%)^§^ | 2,452 (10%)^§^ | 299 (22%)^§^ | 39,814 (16%)^§^ |

Table A1-3. Percent of observed MDRO HAI cases relative to total admissions for patients hospitalized ≥ 2 days. Incidence (number of MDRO HAI over total admissions) is in parentheses. *Percentages are stratified into pre-pandemic (i.e., before 1st SARS-CoV-2-positive patient admission — 224,992 total admissions) and COVID-19 pandemic (i.e., on-or-after 1st SARS-CoV-2-positive patient admission — 25,347 total admissions) periods.

| **HAI** | **Time period^*^** | **Hospital A cases** | **Hospital B cases** | **Hospital C cases** | **Hospital D cases** | **Total cases** |
| --- | --- | --- | --- | --- | --- | --- |
| ***Acinetobacter spp.*** | Pre-pandemic | 0.01%  (11/133,450) | 0.05%  (17/33,820) | 0.01%  (3/53,206) | 0%  (0/4,516) | 0.01%  (31/224,992) |
|  | COVID-19 Pandemic | 0.05%  (8/15,735) | 0.03%  (1/3,825) | 0%  (0/5,466) | 0%  (0/521) | 0.04%  (9/25,547) |
|  |  |  |  |  |  | **0.02%**  **(40/250,539)** |
| ***C. difficile*** | Pre-pandemic | 0.42%  (565/133,450) | 0.22%  (75/33,820) | 0.16%  (86/53,206) | 0.04%  (2/4,516) | 0.32%  (728/224,992) |
|  | COVID-19 Pandemic | 0.52%  (78/15,735) | 0.18%  (6/3,825) | 0.06%  (3/5,466) | 0%  (0/521) | 0.36%  (87/25,547) |
|  |  |  |  |  |  | **0.33%**  **(815/250,539)** |
| ***E. coli*** | Pre-pandemic | 0.25%  (337/133,450) | 0.18%  (60/33,820) | 0.21%  (111/53,206) | 0.4%  (2/4,516) | 0.23%  (510/224,992) |
|  | COVID-19 Pandemic | 0.35%  (52/15,735) | 0.24%  (8/3,825) | 0.15%  (8/5,466) | 0%  (0/521) | 0.28%  (68/25,547) |
|  |  |  |  |  |  | **0.23%**  **(578/250,539)** |
| ***E. faecium*** | Pre-pandemic | 0.15%  (195/133,450) | 0.11%  (37/33,820) | 0.07%  (38/53,206) | 0%  (0/4,516) | 0.12%  (270/224,992) |
|  | COVID-19 Pandemic | 0.15%  (22/15,735) | 0.12%  (4/3,825) | 0.02%  (1/5,466) | 0%  (0/521) | 0.11%  (27/25,547) |
|  |  |  |  |  |  | **0.12%**  **(297/250,539)** |
| ***Enterobacteriaceae* spp.** | Pre-pandemic | 0.50%  (664/133,450) | 0.34%  (116/33,820) | 0.26%  (137/53,206) | 0.04%  (2/4,516) | 0.41%  (919/224,992) |
|  | COVID-19 Pandemic | 0.73%  (110/15,735) | 0.59%  (20/3,825) | 0.33%  (17/5,466) | 0%  (0/521) | 0.61%  (147/25,547) |
|  |  |  |  |  |  | **0.43%**  **(1066/250,539)** |
| ***K. pneumoniae*** | Pre-pandemic | 0.06%  (74/133,450) | 0.07%  (23/33,820) | 0.02%  (8/53,206) | 0%  (0/4,516) | 0.05%  (105/224,992) |
|  | COVID-19 Pandemic | 0.07%  (10/15,735) | 0.12%  (4/3,825) | 0.08%  (4/5,466) | 0%  (0/521) | 0.07%  (18/25,547) |
|  |  |  |  |  |  | **0.05%**  **(123/250,539)** |
| ***P. aeruginosa*** | Pre-pandemic | 0.15%  (198/133,450) | 0.10%  (34/33,820) | 0.04%  (23/53,206) | 0%  (0/4,516) | 0.11%  (255/224,992) |
|  | COVID-19 Pandemic | 0.22%  (33/15,735) | 0.18%  (6/3,825) | 0.08%  (4/5,466) | 0%  (0/521) | 0.18%  (43/25,547) |
|  |  |  |  |  |  | **0.12%**  **(298/250,539)** |
| ***S. aureus*** | Pre-pandemic | 0.24%  (323/133,450) | 0.22%  (73/33,820) | 0.12%  (65/53,206) | 0.02%  (1/4,516) | 0.21%  (462/224,992) |
|  | COVID-19 Pandemic | 0.31%  (46/15,735) | 0.35%  (12/3,825) | 0.08%  (4/5,466) | 0%  (0/521) | 0.26%  (62/25,547) |
|  |  |  |  |  |  | **0.21%**  **(524/250,539)** |

**APPENDIX 2 – MDRO group definitions**

Figure A2-1. If patients in our study had an infection with any *Acinetobacter spp*. pathogen meeting Burnham et al. (2018) definitions, they were classified as MDRO *Acinetobacter*-positive. Table information shown here was first reported in Supplementary Table 1 of:

Burnham JP, Olsen MA, Stwalley D, Kwon JH, Babcock HM, Kollef MH. Infectious diseases consultation reduces 30-day and 1-year all-cause mortality for multidrug-resistant organism infections. *Open forum infectious diseases*. 2018;5(3):ofy026.

Figure A2-2. If patients in our study had an infection with any *Enterobacteriaceae spp*. pathogen meeting Burnham et al. (2018) definitions, they were classified as MDRO *Enterobacteriaceae*-positive. MDRO E. coli and MDRO K. pneumoniae subsets were also identified. Table information shown here was first reported in Supplementary Table 1 of:

Burnham JP, Olsen MA, Stwalley D, Kwon JH, Babcock HM, Kollef MH. Infectious diseases consultation reduces 30-day and 1-year all-cause mortality for multidrug-resistant organism infections. *Open forum infectious diseases*. 2018;5(3):ofy026.

Figure A2-3. If patients in our study had an infection with any *S. aureus* pathogen meeting Burnham et al. (2018) definitions, they were classified as MDRO *S. aureus*-positive. Table information shown here was first reported in Supplementary Table 1 of:

Burnham JP, Olsen MA, Stwalley D, Kwon JH, Babcock HM, Kollef MH. Infectious diseases consultation reduces 30-day and 1-year all-cause mortality for multidrug-resistant organism infections. *Open forum infectious diseases*. 2018;5(3):ofy026.

Figure A2-4. If patients in our study had an infection with any *E. faecium* pathogen meeting Burnham et al. (2018) definitions, they were classified as MDRO *E. faecium*-positive. Table information shown here was first reported in Supplementary Table 1 of:

Burnham JP, Olsen MA, Stwalley D, Kwon JH, Babcock HM, Kollef MH. Infectious diseases consultation reduces 30-day and 1-year all-cause mortality for multidrug-resistant organism infections. *Open forum infectious diseases*. 2018;5(3):ofy026.

Figure A2-4. If patients in our study had an infection with any *P. aeruginosa* pathogen meeting Burnham et al. (2018) definitions, they were classified as MDRO *P. aeruginosa*-positive. Table information shown here was first reported in Supplementary Table 1 of:

Burnham JP, Olsen MA, Stwalley D, Kwon JH, Babcock HM, Kollef MH. Infectious diseases consultation reduces 30-day and 1-year all-cause mortality for multidrug-resistant organism infections. *Open forum infectious diseases*. 2018;5(3):ofy026.

**APPENDIX 3 – Fitting mixed-effect logistic regression models**

*Equations and model selection*

Data spanning the full date range (i.e., Jan. 2017 - Sept. 2020) were used to fit “full time period” models described by the following equation:

$logit(j_{ix}){| \tau}_{ix}=\beta_{0}+\beta_{1}\lambda_{jh\tau_{ix}}+\beta_{2}$COVID-19_period$+ \beta_{3}{Preceding\_LOS}_{j_{ix}}$ ${+ \beta}_{4}{Preceding\_Antibiotic\_Days}_{ix}$ ${+ \beta}_{5}{Preceding\_ICU\_Days}_{ix} + \mu_{h}$,

(Equation 1)

where $j_{ix}$is the binary observation of an HAI associated with a pathogen ($j$) in patient ($i$) during their $x$^th^ observed hospital stay, $\lambda_{jh\tau_{ix}}$ is the cumulative hospital-specific infection pressure – the calculation for which is given in detail below – for pathogen $j$ during the span of patient $i$’s $x$^th^ observed hospital stay ($\tau_{ix}$), and $\mu_{h}$ is a random effect for hospital. We include a single random effect to account for hospital-level variation because preliminary analyses using the Diagnostics for HierArchical Regression Models approach (i.e., DHARMa)^1^ suggested that model errors were not always independent from the hospital variable. The same analyses suggested that model errors were independent from patient IDs ($i$), so no additional random effect was included for this variable.

If the full model failed to converge and generate reliable $\beta$ estimates due to complete/quasi-complete separation, relatively few observed cases, or an overabundance of poorly–fitting observations, then continuous fixed-effects were removed one at a time and reduced models were fit to the data. Because we wanted to keep as many fixed effects as possible, for subsequent analyses we kept the stable model with the greatest degrees of freedom and discarded the rest. We define a “stable model” as one that successfully converged on a maximum-likelihood estimate (MLE), and has a maximum scaled gradient value of < 0.002 at the MLE, as estimated through a combination use of BOBYQA^2^ and Nelder-Mead^3^ optimizers, the default procedure implemented by the “glmer” function in the *lme4* R package.^4^ If >1 stable model existed at the greatest possible degree-of-freedom level, we selected the candidate model with the lowest AIC value. Doing so allowed us to present a model containing as many fixed effects as possible. If the selected stable model had a singular fit, following the guidance given by Oberpriller et al. (2021),^5^ we replaced $\mu_{h}$ with a fixed categorical hospital effect and refit the model to the data. This minimizes Type I error rates associated with model predictions.^3^ Final model forms fit for each HAI are included in Appendix 3. Prior to fitting models to the data, all continuous and non-binary discrete variables to be included in models were rescaled such that they became centered around 0 with standard deviations of 1.

Prior to fitting “COVID-19 time period” models, we further subset our admission data to include only admissions on or after the first day in which a SARS-CoV-2 positive patient was admitted (i.e., 25,547 observations).

$logit(j_{ix}){| \tau}_{ix}=\beta_{0}+\beta_{1}\Delta_{h\tau_{ix}} +\beta_{2}\lambda_{jh\tau_{ix}}+\beta_{3}$COVID-19_diag$\mathrm{nosis}_{ix}$ $+ \beta_{4}{Preceding\_LOS}_{j_{ix}}$ ${+ \beta}_{5}{Preceding\_Antibiotic\_Days}_{ix}$ ${+ \beta}_{6}{Preceding\_ICU\_Days}_{ix} +\mu_{h}$

(Equation 2)

where $\Delta_{h\tau_{ix}}$is the cumulative hospital-specific COVID-19 burden during the span patient $i$’s $x$^th^ hospital stay ($\Delta_{h\tau_{ix}}$ calculation given below). Like with the “full time period” (*see* Equation 1) model set, all continuous and non-binary discrete variables were rescaled and, when necessary, we removed fixed continuous variable effects until a stable model was identified and refit models with singular fits.

*Infection pressure calculations*

Including infection pressure variables in our analyses allows the models to account for time-varying differences in exposure risk, and other indirect risk-modifiers associated with local pathogen prevalence (e.g., redistribution of hospital staff and resources, level of staff adherence to infection control policies, etc.) during each unique hospital stay.^6^ Infection pressure ($\lambda_{jh\tau}$) is estimated as the proportion of patient-days contributed by patients with confirmed cases of the causative pathogen ($j$) in each hospital ($h$) relative to the the total number of patient-days observed in the hospital during any given time period $\tau$.^5^ The value of $\lambda_{jh\tau}$ is given by the equation:

$\lambda_{jh\tau}=\frac{\sum_{i = (1, \ldots, n_{jht})}^{I_{jh\tau}} d_{i\tau}}{\sum_{i = (\ldots... , n_{ht})}^{I_{h\tau}} d_{i\tau}}$,

(Equation 3)

where $I_{h\tau}$ is a vector of unique patient IDs ($i$) for patients observed in hospital $h$ during time period $\tau$, $I_{jh\tau}$ is a subset of $I_{h\tau}$ containing only patient IDs of patients infected with pathogen $j$, and $d_{i\tau}$ is the number of days each patient $i$ was hospitalized during time period $\tau$. Hospital-specific COVID-19 burden ($\Delta_{h\tau}$) is calculated using the same equation. However, for simplicity and because we do not examine the probability of SARS-CoV-2 transmission between hospital patients, we differentiate the infection pressure for HAI pathogens of interest from hospital-level COVID-19 burden by referring to the latter as $\Delta_{h\tau}$. We calculated $\Delta_{h\tau}$ and $\lambda_{jh\tau}$ for HAIs of interest for every time period $\tau_{ijx}$, the time between admittance and HAI-positive test results, or discharge/death of patient $i$ if they had no positive test results, for patient $i$’s $x$^th^ observed hospital stay in our data set.

*Model outcomes*

Table A3-1. Observed beta values for fixed-effect variables in “Full time period” logistic-regression models for predicting hospital-associated infection (HAI) outcomes. Wald 95% confidence intervals are given in parentheses.

| **HAI** | **Variable** | **Beta-value** | ***p*** |
| --- | --- | --- | --- |
| ***Acinetobacter spp.*** | $\beta_{0}$ | -9.60 | - |
|  | $\lambda_{jh\tau_{ix}}$ | -0.46 | 0.04 |
|  | COVID-19_period | 0.97 | 0.01 |
|  | Preceding_LOS | 0.03 | 0.77 |
|  | Preceding_Antibiotic_Days | 0.07 | < 0.001 |
|  | Preceding_ICU_Days | 0.88 | < 0.001 |
| ***C. difficile*** | $\beta_{0}$ | -6.56 | - |
|  | $\lambda_{jh\tau_{ix}}$ | -0.20 | < 0.001 |
|  | COVID-19_period | 0.03 | 0.81 |
|  | Preceding_LOS | 0.10 | < 0.001 |
|  | Preceding_Antibiotic_Days^*^ | - | - |
|  | Preceding_ICU_Days | 0.43 | < 0.001 |
| ***E. coli*** | $\beta_{0}$ | -6.42 | - |
|  | $\lambda_{jh\tau_{ix}}$ | -0.07 | 0.30 |
|  | COVID-19_period | -0.12 | 0.51 |
|  | Preceding_LOS | 0.11 | < 0.001 |
|  | Preceding_Antibiotic_Days | -0.11 | 0.05 |
|  | Preceding_ICU_Days | 0.52 | < 0.001 |
|  | Hospital_1_ | 0 | - |
|  | Hospital_2_ | -0.25 | 0.19 |
|  | Hospital_3_ | -0.03 | 0.81 |
|  | Hospital_4_ | -1.15 | 0.11 |
| ***E. faecium*** | $\beta_{0}$ | -6.92 | - |
|  | $\lambda_{jh\tau_{ix}}$ | -0.08 | 0.36 |
|  | COVID-19_period | -0.06 | 0.78 |
|  | Preceding_LOS | 0.18 | < 0.001 |
|  | Preceding_Antibiotic_Days | 0.15 | < 0.001 |
|  | Preceding_ICU_Days | 0.24 | < 0.001 |
| ***Enterobacteriaceae* spp.** | $\beta_{0}$ | -6.32 | - |
|  | $\lambda_{jh\tau_{ix}}$ | -0.15 | 0.002 |
|  | COVID-19_period | 0.23 | 0.05 |
|  | Preceding_LOS | 0.12 | < 0.001 |
|  | Preceding_Antibiotic_Days | 0.03 | 0.23 |
|  | Preceding_ICU_Days | 0.62 | < 0.001 |
| ***K. pneumoniae*** | $\beta_{0}$ | -8.18 | - |
|  | $\lambda_{jh\tau_{ix}}$ | -0.03 | 0.82 |
|  | COVID-19_period | -0.27 | 0.46 |
|  | Preceding_LOS | 0.11 | < 0.001 |
|  | Preceding_Antibiotic_Days | 0.06 | 0.04 |
|  | Preceding_ICU_Days | 0.60 | < 0.001 |
| ***P. aeruginosa*** | $\beta_{0}$ | -7.62 | - |
|  | $\lambda_{jh\tau_{ix}}$ | -0.22 | 0.03 |
|  | COVID-19_period | 0.37 | 0.04 |
|  | Preceding_LOS | 0.15 | < 0.001 |
|  | Preceding_Antibiotic_Days | 0.13 | 0.007 |
|  | Preceding_ICU_Days | 0.74 | < 0.001 |
| ***S. aureus*** | $\beta_{0}$ | -6.78 | - |
|  | $\lambda_{jh\tau_{ix}}$ | 0.09 | 0.14 |
|  | COVID-19_period | 0.05 | 0.73 |
|  | Preceding_LOS | 0.05 | 0.04 |
|  | Preceding_Antibiotic_Days | 0.10 | 0.004 |
|  | Preceding_ICU_Days | 0.76 | < 0.001 |

Table A3-2. Observed beta values for fixed-effect variables in “COVID-19 time period” logistic-regression models for predicting hospital-associated infection (HAI) outcomes.

| **HAI** | **Variable** | **Beta-value** | ***p*** |
| --- | --- | --- | --- |
| ***Acinetobacter spp.*** | $\beta_{0}$ | -9.03 | - |
|  | $\lambda_{jh\tau_{ix}}$ | -0.67 | 0.14 |
|  | $\Delta_{h\tau_{ix}}$ | 0.12 | 0.57 |
|  | COVID-19_diagnosis | 0.25 | 0.83 |
|  | Preceding_LOS | 0.03 | 0.88 |
|  | Preceding_Antibiotic_Days | 01.69 | < 0.001 |
|  | Preceding_ICU_Days | 0.89 | < 0.001 |
|  | Hospital_1_ | 0 | - |
|  | Hospital_2_ | -1.55 | 0.32 |
|  | Hospital_3_ | -17.47 | 0.99 |
|  | Hospital_4_ | -16.41 | 1.00 |
| ***C. difficile*** | $\beta_{0}$ | -6.28 | - |
|  | $\lambda_{jh\tau_{ix}}$ | -0.23 | 0.34 |
|  | $\Delta_{h\tau_{ix}}$ | -0.13 | 0.20 |
|  | COVID-19_diagnosis | 0.07 | 0.89 |
|  | Preceding_LOS | 0.14 | 0.04 |
|  | Preceding_Antibiotic_Days^*^ | 0.53 | < 0.001 |
|  | Preceding_ICU_Days | 0.41 | < 0.001 |
| ***E. coli*** | $\beta_{0}$ | -6.77 | - |
|  | $\lambda_{jh\tau_{ix}}$ | 0.00 | 0.99 |
|  | $\Delta_{h\tau_{ix}}$ | 0.08 | 0.49 |
|  | COVID-19_diagnosis | 0.34 | 0.55 |
|  | Preceding_LOS | 0.20 | 0.008 |
|  | Preceding_Antibiotic_Days | -0.21 | 0.27 |
|  | Preceding_ICU_Days | 0.52 | < 0.001 |
|  | Hospital_1_ | 0 | - |
|  | Hospital_2_ | -0.83 | 0.32 |
|  | Hospital_3_ | -0.52 | 0.39 |
|  | Hospital_4_ | -13.70 | 0.99 |
| ***E. faecium*** | $\beta_{0}$ | -6.77 | - |
|  | $\lambda_{jh\tau_{ix}}$ | 0.13 | 0.66 |
|  | $\Delta_{h\tau_{ix}}$ | 0.00 | 1.00 |
|  | COVID-19_diagnosis | 0.26 | 0.75 |
|  | Preceding_LOS | 0.25 | 0.005 |
|  | Preceding_Antibiotic_Days | 0.95 | < 0.001 |
|  | Preceding_ICU_Days | 0.28 | 0.03 |
|  | Hospital_1_ | 0 | - |
|  | Hospital_2_ | -0.52 | 0.50 |
|  | Hospital_3_ | -1.61 | 0.13 |
|  | Hospital_4_ | -0.14 | 0.99 |
| ***Enterobacteriaceae* spp.** | $\beta_{0}$ | -5.36 | - |
|  | $\lambda_{jh\tau_{ix}}$ | 0.00 | 1.00 |
|  | $\Delta_{h\tau_{ix}}$ | -0.03 | 0.58 |
|  | COVID-19_diagnosis | 0.56 | 0.05 |
|  | Preceding_LOS | 0.19 | < 0.001 |
|  | Preceding_Antibiotic_Days | -0.21 | 0.03 |
|  | Preceding_ICU_Days | 0.51 | < 0.001 |
|  | Hospital_1_ | 0 | - |
|  | Hospital_2_ | -0.18 | 0.60 |
|  | Hospital_3_ | -0.54 | 0.09 |
|  | Hospital_4_ | -0.13 | 0.96 |
| ***K. pneumoniae*** | $\beta_{0}$ | -8.29 | - |
|  | $\lambda_{jh\tau_{ix}}$ | -1.87 | 0.04 |
|  | $\Delta_{h\tau_{ix}}$ | -0.11 | 0.60 |
|  | COVID-19_diagnosis | 0.23 | 0.84 |
|  | Preceding_LOS | 0.11 | 0.53 |
|  | Preceding_Antibiotic_Days | -0.10 | 0.79 |
|  | Preceding_ICU_Days | 0.61 | 0.004 |
|  | Hospital_1_ | 0 | - |
|  | Hospital_2_ | -0.09 | 0.94 |
|  | Hospital_3_ | -0.91 | 0.43 |
|  | Hospital_4_ | -14.92 | 1.00 |
| ***P. aeruginosa*** | $\beta_{0}$ |  | - |
|  | $\lambda_{jh\tau_{ix}}$ | -0.82 | 0.008 |
|  | $\Delta_{h\tau_{ix}}$ | -0.15 | 0.19 |
|  | COVID-19_diagnosis | 0.80 | 0.08 |
|  | Preceding_LOS | 0.15 | 0.03 |
|  | Preceding_Antibiotic_Days | 0.47 | 0.009 |
|  | Preceding_ICU_Days | 0.73 | < 0.001 |
|  | Hospital_1_ | 0 | - |
|  | Hospital_2_ | -0.53 | 0.44 |
|  | Hospital_3_ | -1.52 | 0.03 |
|  | Hospital_4_ | -15.00 | 0.99 |
| ***S. aureus*** | $\beta_{0}$ | -6.95 | - |
|  | $\lambda_{jh\tau_{ix}}$ | 0.03 | 0.76 |
|  | $\Delta_{h\tau_{ix}}$ | -0.04 | 0.83 |
|  | COVID-19_diagnosis | 1.43 | < 0.001 |
|  | Preceding_LOS | -0.16 | 0.21 |
|  | Preceding_Antibiotic_Days | 0.15 | 0.34 |
|  | Preceding_ICU_Days | 0.70 | < 0.001 |

REFERENCES

1. Hartig F. DHARMa: residual diagnostics for hierarchical (multi-level / mixed) regression models. R package ver 0.4.5. 2022.

2. Powell MJD. The BOBYQA algorithm for bound constrained optimization without derivatives. Technical Report, Department of Applied Mathematics and Theoretical Physics. 2009.

3. Nelder JA, Mead R. A simplex method for function minimization. *The Computer Journal*. 1965;7(4):308-313.

4. Bates D, Mächler M, Bolker B, Walker S. Fitting linear mixed-effects models using lme4. *J Stat Soft*. 2015;67(1):1.

5. Oberpriller J, De Souza Leite M, Pichler M. Fixed or random? on the reliability of mixed-effects models for a small number of levels in grouping variables. Preprint. 2021. doi: 10.1101/2021.05.03.442487.

6. Ajao AO, Harris AD, Roghmann M, et al. Systematic review of measurement and adjustment for colonization pressure in studies of methicillin-resistant staphylococcus aureus, vancomycin-resistant enterococci, and clostridium difficile acquisition. *Infection Control & Hospital Epidemiology*. 2011;32(5):481-489.
